# Dual role of Lyz2-positive myeloid cells in traumatic brain injury: acute anti-inflammatory effects vs. chronic neurological deterioration

**DOI:** 10.3389/fncel.2025.1642410

**Published:** 2025-10-07

**Authors:** Hua-Zheng Yan, Yi-Wan Fang, Shi-Yu Zhou, Jian-Xiong Gao, Ming-Ming Bian, Yao-Mei Xu, Lin Zhang, Nan Zhang, He-Zuo Lü

**Affiliations:** 1 Clinical Laboratory, The First Affiliated Hospital of Bengbu Medical University, Bengbu, Anhui, China; 2 Anhui Key Laboratory of Tissue Transplantation, The First Affiliated Hospital of Bengbu Medical University, Bengbu, Anhui, China; 3 Anhui Province Key Laboratory of Basic and Translational Research of Inflammation-related Diseases, Bengbu, Anhui, China; 4 Department of Immunology, Bengbu Medical University, Bengbu, Anhui, China

**Keywords:** traumatic brain injury, neuroinflammation, myeloid cell polarization, myeloid cell polarization functional recovery, acute-chronic neuroimmunology

## Abstract

**Introduction:**

Neuroinflammation is a critical factor contributing to secondary brain injury following traumatic brain injury (TBI). This process engages diverse cell types within the central nervous system (CNS), including significant infiltration of myeloid lineage cells–primarily neutrophils and macrophages–during the acute and subacute phases of TBI. These myeloid-derived cells represent a major population that critically influences the development and progression of neuroinflammation. Microglia and peripherally infiltrating macrophages exhibit polarization phenotypes that play a pivotal role in modulating inflammatory changes. Due to their functional and phenotypic similarities, their distinct contributions to the inflammatory response in TBI remain a subject of considerable debate. Lysozyme 2 (Lyz2) is a well-established marker for myeloid lineage cells (including monocytes, macrophages, and neutrophils) in mice, allowing specific targeting and depletion of these cells to dissect their functional roles in TBI.

**Methods:**

In the present study, we investigated the trend of inflammatory factors during the early stage of TBI using Lyz2-IRES-DTREGFP transgenic mice, which specifically target and deplete Lyz2-positive myeloid cells. Tissue samples for RT-qPCR and flow cytometry were harvested from the perilesional cortex (within a 2-mm radius of the impact site) and the underlying hippocampus.

**Results and discussion:**

Our findings revealed a considerable reduction in the expression of pro-inflammatory factors (e.g., IL-1β, iNOS, IL-6, IFN-γ) and an increase in the expression of anti-inflammatory factors (e.g., IL-4, IL-10, IL-13, Arg-1). Furthermore, we observed a shift in polarization phenotypes, characterized by a decreased proportion of M1 macrophages and an increased proportion of M2 macrophages. However, during the chronic phase, behavioral and histological analyses revealed worse outcomes. These findings demonstrate that targeted depletion of Lyz2-positive myeloid cells during acute TBI attenuates neuroinflammation. However, this early immunomodulatory shift correlates paradoxically with exacerbated chronic neurological deficits, suggesting that transient suppression of myeloid-driven inflammation may disrupt long-term reparative processes critical for functional recovery after TBI.

## Introduction

Traumatic brain injury (TBI) is a major contributor to death and disability worldwide. The burden of TBI is particularly pronounced in low- and middle-income countries, where morbidity and mortality rates are significantly higher, imposing substantial economic costs on both affected individuals and the global economy (Khellaf et al., 2019). Usually, TBI is divided into primary and secondary traumas. Primary injury refers to the direct damage to brain tissue caused by external mechanical forces, such as crushing, impact, or penetrating trauma. These forces often result in axonal disruption, contusions, and hemorrhage formation (Lee et al., 2019). The primary injury often progresses into a secondary injury, which is characterized by a complex physiological and biochemical cascade. This cascade primarily involves ischemia, oxidative stress, and neuroinflammation, ultimately contributing to long-term brain damage (Thapa et al., 2021). Due to the inevitability of primary injury and the prolonged nature of secondary injury, which can persist for months or even years, secondary injury represents a critical target for therapeutic intervention in TBI. However, despite its potential, no highly effective treatments or drugs targeting this phase have yet been successfully translated into clinical practice (Sulhan et al., 2018). Therefore, understanding the interactions between various molecules and cells following secondary injury represents a highly feasible approach to mitigating its effects. By targeting and modulating harmful immune responses, it may be possible to prevent or reduce the progression of secondary injury.

Myeloid cells constitute the primary cellular component of the innate immune system and play a critical role in the response to central nervous system (CNS) injury. Broadly defined, myeloid cells encompass granulocytes, macrophages, monocytes, and dendritic cells (Kumar et al., 2016). Immediately following CNS injury, the disruption of blood-brain barrier integrity triggers a state of systemic immunosuppression. This disruption facilitates the release of damage-associated molecular patterns (DAMPs) and alarmins, which promote the recruitment of peripheral immune cells to the injury site (Wilson et al., 2010). The first responders to CNS injury are myeloid cells, among which neutrophils rapidly accumulate at the injury site within hours to days following the initial trauma (Liu et al., 2018). Microglia rapidly transition to an activated state and proliferate extensively within days following injury. Subsequently, monocytes infiltrate the injured CNS, where they differentiate into macrophages and persist to phagocytose cellular debris (Devanney et al., 2020). They are also a source of pro-inflammatory cytokines and nitric oxide, exacerbating damage to the CNS (Kozlov et al., 2017). Current evidence indicates that microglia originate from erythromyeloid precursor cells in the yolk sac during early embryonic development, highlighting their classification within the myeloid lineage (You et al., 2023). And activated microglia undergo significant morphological changes and are difficult to distinguish from peripherally infiltrating macrophages (Trahanas et al., 2015). Moreover, microglia and peripherally infiltrating macrophages share numerous identical or similar markers, rendering them difficult to distinguish using conventional morphological or immunological techniques. These cells make up the majority of immune cell populations during the acute and subacute stages of TBI, yet their overlapping characteristics complicate their differentiation. Consequently, their respective roles in the inflammatory response to TBI remain a subject of significant debate. Lysozyme M (LysM), which is encoded by the *Lyz2* gene, is widely expressed in monocytes, macrophages, and neutrophils. Owing to this specific expression profile, it has been extensively used as a reliable marker for murine myeloid cells (Blank and Prinz, 2016). Although the roles of myeloid cells are complex and sometimes controversial, their core functions primarily involve “clearance” and “signaling”. For instance, in ischemic injury, detrimental myeloid cell populations can exacerbate inflammation and cell death within the affected area, thereby impairing angiogenesis and neurogenesis (Anthony et al., 2022). Similarly, in neurodegenerative diseases, harmful myeloid subsets can sustain chronic inflammation—driving disease progression—and exhibit impaired phagocytic capacity, failing to effectively clear pathogenic proteins (Gao et al., 2023). Beneficial populations, on the other hand, help maintain neuronal homeostasis and support synaptic function (Colonna, 2017).

With the advancement and refinement of gene editing technologies, genetically modified mice have become indispensable tools in biomedical research. Techniques such as gene knockout, conditional gene overexpression, and transgenic modifications at the gene level are now widely employed. In earlier studies, a mouse model was successfully developed to specifically express enhanced green fluorescent protein (EGFP) in myeloid lineage cells (Faust et al., 2000). By fusing the diphtheria toxin receptor (DTR) with enhanced green fluorescent protein (EGFP) and integrating the DTREGFP construct into the Lyz2 locus of mice via homologous recombination, a Lyz2-IRES-DTREGFP mice was generated that enables specific expression of DTR in myeloid cells. Subsequently, intraperitoneal injection of diphtheria toxin (DT) induces cell-specific ablation, as DT binds to the DTR and exerts cytotoxic effects, leading to targeted cell death (Goren et al., 2009). In this study, we employed Lyz2-IRES-DTREGFP mice to investigate the temporal duality of Lyz2-positive myeloid cells in a controlled cortical impact (CCI) model of TBI. We focused on the acute (1-7 days) and chronic (28 days) phases, analyzing cytokine profiles, polarization states, and functional recovery.

## Materials and methods

### Animal

A total of 186 specific pathogen-free (SPF) grade, healthy adult Lyz2-IRES-DTREGFP female mice (aged 8 weeks, weighing 20-23 g) were used in this study (Table 1). The mice were obtained from Shanghai Southern Model Biotechnology Co., Ltd., with the animal license number SCXK (Shanghai) 2017-0010. Every experimental process and animal care was carried out strictly in accordance with the regulations set forth by China's Ministry of Science and Technology. All experimental groups were sent by random number table.

**Table 1:** Total number of mice used in this study.

**Experiment**	**Animal number**
Flow cytometry	54
Quantitative real-time PCR (RT-qPCR)	36
Immunohistofluorescence	36
Histology and behavior	60
Total	186

### Mouse genotyping

Mouse toe tissue was collected and placed in a 1.5 mL nuclease-free microcentrifuge tube. Then, 200 μL of protease lysis buffer (containing Proteinase K and 1 × Mouse Tissue Lysis Buffer; TIANGEN, Beijing, China) was added. After complete lysis, the upper aqueous phase containing RNA was collected for reverse transcription. The resulting cDNA was diluted with DEPC-treated water to a concentration ranging from 1 to 10 ng/μL. The PCR reaction mixture was prepared on ice according to the manufacturer's instructions, using the following cycling conditions: initial denaturation at 94 °C for 5 minutes; 35 cycles of denaturation at 94 °C for 30 seconds, annealing at 55 °C for 30 seconds, and extension at 72 °C for 30 seconds; followed by a final extension at 72 °C for 7 minutes. The primer sequences used in this study are listed in Table 2.

**Table 2:** Primer sequences.

**Omnipotent**	**Primer**	**Primer sequence (5′ → 3′)**	**Primer type**	**Product length/bp**		
				**C57BL/6J**	**Lyz2-EGFP-DTR** ^+/−^	**yz2-EGFP-DTR** ^+/+^
Lyz2-EGFP-DTR	P1	GAGCACACTGTCAAAACCGAGAT	Forward	326 bp	326 bp	–
	P2	TCCTGGCTGAAGAACTGACCTAC	Reverse
	P3	TTCTTCAAGGACGACGGCAACTA	Forward	–	512 bp	512 bp
	P4	GAGGGGAAATCGAGGGAATGTGAC	Reverse

### Diphtheria toxin (DT) administration

DT (List Labs, USA) was diluted to 0.15 mg/ml in saline and stored at −80 °C. Prior to use, the toxin was administered via intraperitoneal injection at a dose of 25 ng per gram of body weight in saline for three consecutive days before CCI surgery. All injections were performed at the same time each day.

### Controlled cortical impact injury (CCI) mouse model

The mice were anesthetized with a cocktail of ketamine (80 mg/kg)/xylazine (10 mg/kg) intraperitoneally injection. A mouse brain stereotaxic apparatus (Reward, China) was used to precisely locate the hippocampal region (AP: −1.7 mm, ML: −1.2 mm, DV: −2.0 mm). After localization, a circular craniotomy was performed on the skull surface using a drill. The mice were then positioned on a PSI-IH impactor (PSI, USA), and an impact force of 100 kDynes was delivered to induce a moderate brain contusion. Successful modeling was confirmed by the appearance of a red impact site on the cerebral surface that gradually expanded and was accompanied by slight hemorrhaging. Both male and female mice were included and randomly assigned to four groups: (1) the Sham group, which underwent craniotomy without contusion injury; (2) the Sham+DT group, which received DT injections for three consecutive days prior to the experiment, with other procedures identical to the Sham group; (3) the TBI group, which underwent contusion injury; and (4) the DT+TBI group, which received DT injections for three consecutive days prior to the experiment followed by contusion injury.

### Flow cytometry (FCM)

The mice were anesthetized as above. A volume of 50 μL of blood was collected from the mouse tail vein and mixed with 1 mL of erythrocyte lysis buffer (ThermoFisher, USA). Following two rounds of washing with phosphate-buffered saline (PBS; Biosharp, China), the sample was preserved by fixing it with 2% paraformaldehyde (PFA; Biosharp, China). Following PBS perfusion, the liver, lung, spleen, bone marrow, and brain were harvested from the mice. Tissues were prepared in accordance with the guidelines provided by the manufacturer. (Reward, China). After being homogenized and centrifuged, the liver, lung, and spleen were twice cleaned with PBS and preserved with 2% PFA. A syringe was used to clean away bone marrow cells after the femur was removed and both ends were severed. After two PBS washes, the cells were fixed with 2% PFA. Brain tissue was homogenized and centrifuged, followed by fixation, permeabilization, and incubation with antibodies at 4 °C for 30 minutes. After washing with PBS to remove unbound antibodies, the samples were fixed with 2% PFA. The antibodies used in this study are listed in Table 3.

**Table 3:** Antibodies used in the study.

**Antige**	**Host species and clone**	**Cat. # or Lot#**	**RRID**	**Conjugation**	**Source**	**Used concentration**	**Methods**
CD11b	Rat monoclonal	130-113-238		VioBlue	Miltenyi Biotec	1:50	FCM
CD45	Rat monoclonal	17-0451-82	AB_469392	APC	Invitrogen	0.125 μg/test	
CD68	Rat monoclonal	61-0681-82	AB_2802375	PE-eFluor™ 610	Invitrogen	0.2 mg/mL	
CCR7	Rat monoclonal	47-1971-82	AB_2573974	APC-eFluor™ 780	Invitrogen	0.2 mg/mL	
Arg-1	Rat monoclonal	46-3697-82	AB_2734843	PerCP-eFluor™ 710	Invitrogen	0.2 mg/mL	
CD68	mouse monoclonal	MA5–13324	AB_10987212	Unconjugated	Invitrogen	1: 200	IHF
Arg-1	rabbit polyclonal	PA5–29645	AB_2547120	Unconjugated	Invitrogen	1: 200	
CCR7	Rabbit monoclonal	ab32527	AB_726208	Unconjugated	Abcam	1: 200	
Rabbit IgG (H + L)	donkey polyclonal	711–025-152	AB_2340588	Rhodamine (TRITC)	Abcam	1: 200	
Rabbit IgG (H + L)	goat polyclonal	SA00014-9		CoraLite^®^647	Proteintech	1: 200	

### Reverse transcription-quantitative polymerase chain reaction (RT-qPCR)

Three days post-CCI, mice were anesthetized as above and perfused with 15 mL of phosphate-buffered saline (PBS; Biosharp, China) via cardiac injection. After perfusion, the skull was opened, and the region of interest, which was around 2 mm of tissue surrounding the damage site, was put in an EP tube devoid of enzymes. Trizol reagent (Ambion, USA) was used to extract total RNA from the tissue, and a commercial kit was then used to reverse-transcribe the RNA into cDNA. Taq SYBR^®^ Green qPCR Premix (Best Enzymes, Jiangsu Bestime, China) was used for quantitative real-time PCR (qPCR) on a QuantStudioTM 3 PCR Detection System (Applied Biosystems, CA, USA). Relative gene expression levels were calculated using the Log2 FC method (Taylor et al., 2019; Bustin et al., 2009; Livak, 2001). To determine the relative expression of the genes in each group, the Sham groups were normalized. Table 4 lists the primer sequences utilized in this investigation.

**Table 4:** RT-qPCR Primer sequences.

**Gene**	**Forward primer 5^′^-3^′^**	**Reverse primer 5^′^-3^′^**
GAPDH	GGTTGTCTCCTGCGACTTCA	TGGTCCAGGGTTTCTTACTCC
IL-1β	GCAACTGTTCCTGAACTCAACT	ATCTTTTGGGGTCCGTCAACT
iNOS	GTTCTCAGCCCAACAATACAAGA	GTGGACGGGTCGATGTCAC
IL-6	CCAAGAGGTGAGTGCTTCCC	CTGTTGTTCAGACTCTCTCCCT
IL-13	CCTGGCTCTTGCTTGCCTT	GGTCCTGTGTGATGTTGCTCA
IFN-γ	ATGAACGCTACACACTGCATC	CCATCCTTTTGCCAGCTAGT
IL-4	GGTCTCAACCCCCAGCTAGT	GCCGATGATCTCTCTCAAGTGAT
IL-10	GCTCTTACTGACTGGCATGAG	CGCAGCTCTAGGAGCATGTG
Arg-1	CTCCAAGCCAAAGTCCTTAGAG	AGGAGCTGTCATTAGGGACATC

### Immunohistofluorescence (IHF)

On the seventh day post-CCI, mice were anesthetized as above and perfused with 15 mL of phosphate-buffered saline (PBS; Biosharp, China) followed by 15 mL of 4% paraformaldehyde (PFA; Biosharp, China). After perfusion, the skull was opened, and the brain was carefully removed. The harvested brain tissues were fixed by immersion in 4% PFA for 24 hours, followed by dehydration in 20% and 30% sucrose solutions (Macklin, Canada) for 24 hours each. After dehydration, the tissues were embedded in optimal cutting temperature (OCT) compound (Jiangsu Kangcheng Bai'ao, China) and sectioned into 10 μm slices using a cryostat (Leica, Germany). For immunofluorescence staining, tissue sections on slides were first incubated with primary antibody overnight at 4 °C. The next day, unbound primary antibody was washed off with PBS, and then the sections were treated for one and a half hours at 37 °C with secondary antibody. The sections were coverslipped and mounted with glycerol containing Hoechst after being washed with PBS to eliminate the secondary antibody. A ZEISS Axio Observer microscope (Carl Zeiss, Oberkochen, Germany) was used for imaging.

### Behavioral tests

The open-field test was conducted 21 days post-CCI. Mice were transported to the testing room two hours prior to the experiment to allow acclimatization to the environment. The testing room was maintained under quiet conditions with low, uniform lighting to ensure consistent video recording quality. Each mouse began the experiment in a 50 × 50 × 30 cm open-field arena, and its movement was recorded for 10 minutes to analyze the route it followed and the duration of time it spent in the middle of the arena. The arena was meticulously cleansed to get rid of pee and feces after every trial, wiped with alcohol, and dried to eliminate residual odors that could influence the behavior of subsequent mice (Griffiths et al., 2019).

To assess the mice's cognitive memory ability, the new object recognition test was administered 22 days after CCI. This test calculates how much time the animals spend investigating a known object as opposed to a new one, providing an index of recognition memory (Xue et al., 2024). Mice were transported to the testing room 2 h prior to the experiment to allow acclimatization to the environment. The testing room was maintained under quiet conditions with dim, uniform lighting to ensure consistent video recording quality. At the start of the experiment, two identical cylinders (A and B) were placed at opposite ends of the same side of a white arena. Each mouse was then placed in the arena, and its exploration time of the two objects was recorded over a 10-min period. At the end of the first recording, the mice were taken out and the cylinder A was replaced with a new rectangular object (C) before being put back into the mice, and then the mice were recorded exploring both objects for a further 10 min. The results were analyzed to assess recognition memory. To avoid lingering smells affecting further testing, the arena and items were carefully cleaned with alcohol, dried, and reset in between trials.

Between days 23 and 27 after CCI, the mice's spatial learning and memory skills were assessed using the Morris water maze test. This test evaluates the animals' ability to use spatial clues to navigate and recall the location of a hidden platform (Henry et al., 2020). The pool was filled with water, and a heater was used to maintain the water temperature at approximately 22 °C. The platform was placed in the second quadrant, and mice were introduced into the water from the fourth quadrant. The escape latency was defined as the time from when the mouse entered the water to when it located the platform and remained on it for at least 5 s. If a mouse failed to find the platform within 60 s, it was gently guided to the platform, and the escape latency was recorded as 60 s. After each trial, mice were dried to prevent hypothermia. During the first 4 days, mice underwent two training sessions per day, with an interval of at least 30 min between sessions. On the fifth day, the platform was removed, and a spatial probe test was conducted to assess memory retention. Escape latency, the number of platform crossings, and the time spent in the target quadrant were recorded and analyzed. Prior to modeling, all mice were pre-trained to exclude those with poor swimming ability or performance.

### Histological analysis

On day 28 post-CCI, mice were perfusion-fixed, and brain tissue sections of 10 μm thickness were prepared. The sections were stained with hematoxylin and eosin (HE; Solarbio, China), Nissl staining (Solarbio, China), and Luxol Fast Blue (LFB; Solarbio, China) according to the manufacturer's protocols. The stained sections were analyzed to quantify the injury area, the number of surviving neurons, and the relative optical density (OD) values. Data were statistically analyzed using ImageJ software.

### Statistical analysis

Statistical analyses were performed using GraphPad Prism 9.0 (GraphPad Software, San Diego, CA, USA). Data are presented as mean ± standard error of the mean (SEM). The data from Morris water maze training (escape latency) was reanalyzed using a two-way analysis of variance (ANOVA). The other data were analyzed using nonparametric Kruskal-Wallis analysis of variance. *P* < 0.05 was considered statistically significant.

## Results

### Characterization and genotypic validation of Lyz2-IRES-DTREGFP mice

**Figure 1:**
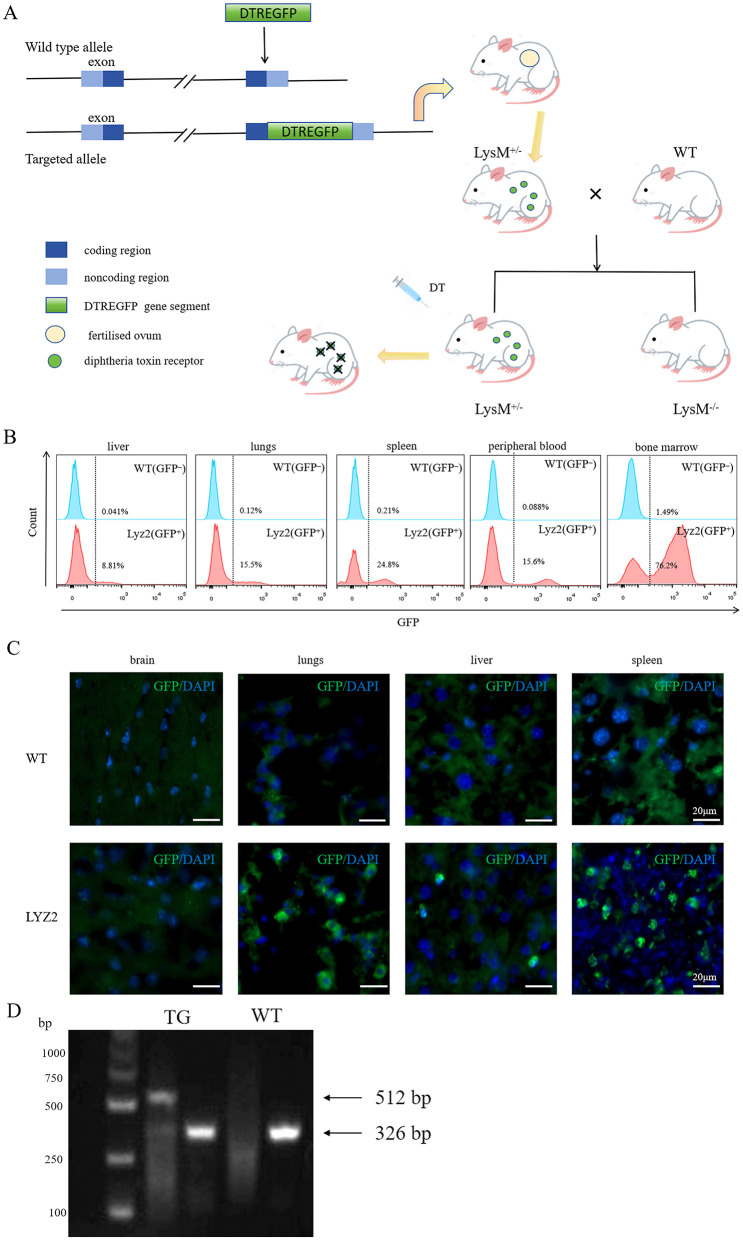
Characterization and genotypic validation of Lyz2-IRES-DTREGFP mice. **(A)**: Schematic of the Lyz2-IRES-DTREGFP transgenic construct design. **(B)**: Flow cytometry histogram showing GFP^+^ cell distribution in liver, spleen, lung, peripheral blood, and bone marrow of Lyz2-IRES-DTREGFP mice versus wild-type (WT) controls. GFP^+^ cells were predominantly detected in peripheral blood, bone marrow, and spleen, with minimal expression in lung and liver. **(C)**: Immunofluorescence staining of GFP^+^ cells (green) in spleen, lung, and liver tissues. No GFP^+^ cells were observed in brain sections. Nuclei were counterstained with DAPI (blue). **(D)**: Genotyping results via PCR and agarose gel electrophoresis. Transgenic mice (TG) exhibited dual DNA fragments (512 bp and 326 bp), while WT mice (WT) showed only the 326 bp fragment.

The Lyz2-IRES-DTREGFP mice were generated by crossing strategies as shown in Figure 1A. To validate GFP^+^ cell specificity in Lyz2-IRES-DTREGFP mice, flow cytometry was performed on tissues from wild-type (WT) and transgenic mice. GFP^+^ cells were predominantly detected in peripheral blood, bone marrow, and spleen, with minimal expression in lungs and liver (Figure 1B, *n* = 3). Immunofluorescence staining detected GFP^+^ cells in lung, liver, and spleen tissues, but not in the brain (Figure 1C), which is consistent with the flow cytometry results. Genotyping via PCR and agarose gel electrophoresis identified transgenic mice by dual 512 bp and 326 bp DNA fragments, whereas WT mice exhibited only the 326 bp fragment (Figure 1D).

### DT-mediated ablation of GFP^+^ myeloid cells in Lyz2-IRES-DTREGFP mice

**Figure 2:**
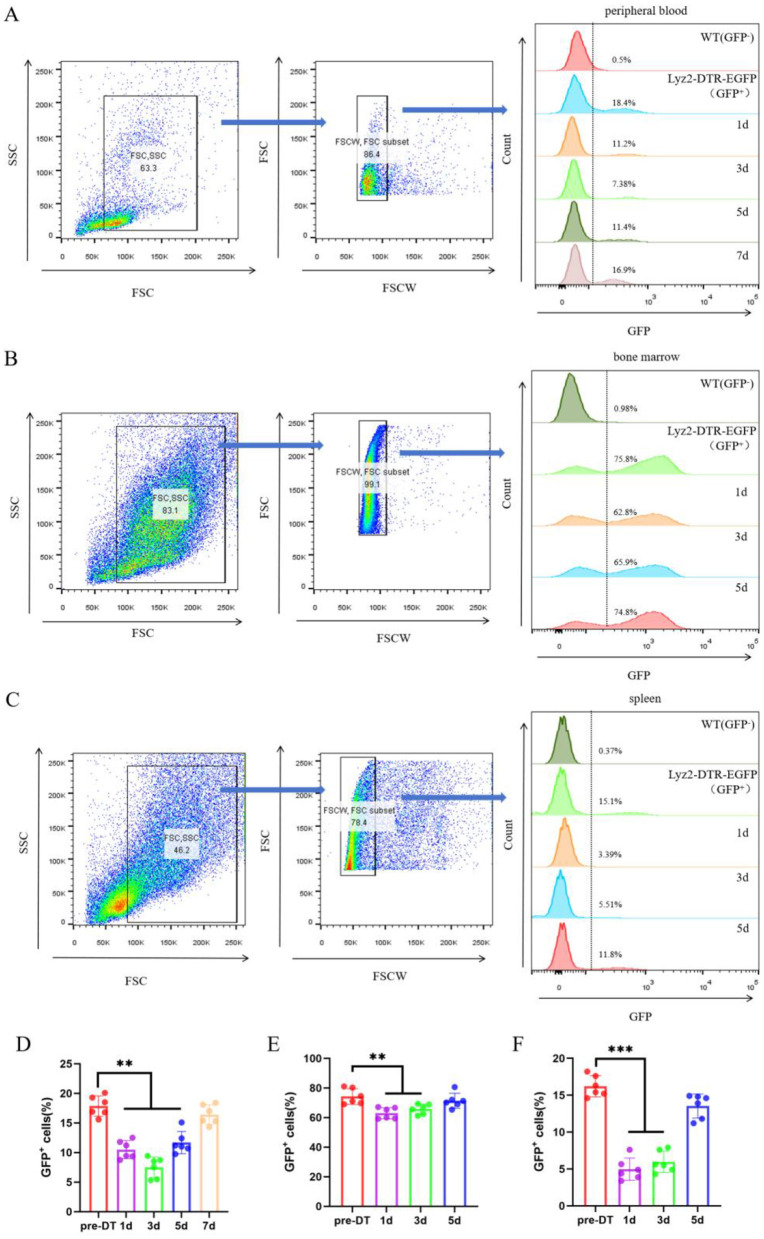
DT-Mediated Ablation of GFP^+^ Myeloid Cells in Lyz2-IRES-DTREGFP Mice. **(A–C)** Pseudocolor flow cytometry plots illustrate the ablation of GFP-positive myeloid populations in peripheral blood, bone marrow, and spleen following a 3-day course of diphtheria toxin (DT; 25 ng/g daily). The depletion kinetics show that GFP^+^ cells in peripheral blood reached their lowest level on day 3, while those in bone marrow and spleen were maximally depleted on day 1. Partial recovery was evident by day 5 (blood) and day 3 (bone marrow/spleen). Wild-type (WT) mice were used as negative controls. **(D–F)** Quantitative analysis of the time course of GFP^+^ cell depletion in peripheral blood **(D)**, bone marrow **(E)**, and spleen **(F)**. Data are represented as mean ± SEM (n = 6). *P < 0.05, **P < 0.01, ***P < 0.001 vs. baseline (pre-DT).

To specifically ablate myeloid cells in vivo, we employed Lyz2-IRES-DTREGFP transgenic mice and administered DT (25 ng/g for 3 consecutive days). Flow cytometric analysis revealed a rapid and robust depletion of GFP^+^ cells across all examined tissues following DT injection (Figures 2A–C). Quantification of this effect (Figures 2D–F) demonstrated a sharp decline in the frequency of GFP^+^ myeloid cells as early as day 1 post-injection. The depletion reached its nadir at day 3 in the peripheral blood (Figure 2D), and even more rapidly at day 1 in both the bone marrow and spleen (Figures 2E, F). Notably, this ablation was transient, as the GFP^+^ cell population began to recover and showed significant reconstitution by days 5 or 7 across all compartments, although it did not fully return to baseline levels within this timeframe. No significant changes in GFP^+^ cell frequencies were observed in DT-treated wild-type control mice (data not show), confirming the specific and genetically targeted nature of the ablation. Together, these data demonstrate that our DT-based system enables efficient, rapid, and reversible depletion of myeloid cells in Lyz2-IRES-DTREGFP mice.

### Myeloid depletion attenuates early pro-inflammatory signaling

**Figure 3:**
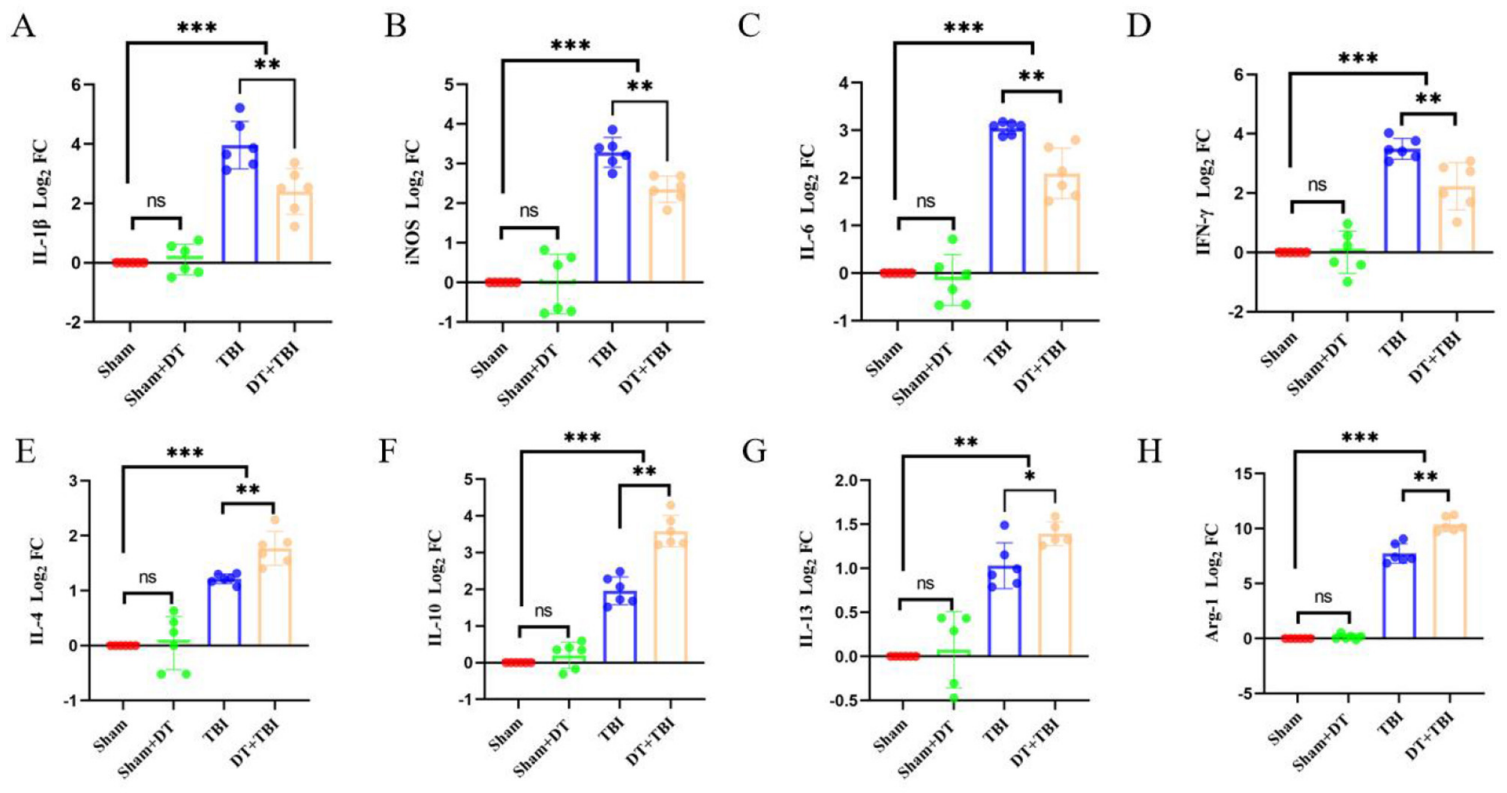
Myeloid depletion attenuates pro-inflammatory signaling and enhances anti-inflammatory mediators post-CCI. A-H: RT-qPCR analysis of pro-inflammatory (IL-1β, iNOS, IL-6, IFN-γ) and anti-inflammatory (IL-4, IL-10, IL-13, Arg-1) cytokine Log_2_ FC value in brain tissue 3 days post-CCI, normalized to GAPDH. Sham (uninjured controls), TBI (CCI alone), and DT+TBI (CCI with myeloid depletion) groups were compared. DT+TBI mice exhibited reduced pro-inflammatory **(A–D)** and elevated anti-inflammatory **(E–H)** cytokine expression versus TBI mice. Data are mean ± SD (*n* = 6); *P < 0.05, **P < 0.01, ***P < 0.001 (DT+TBI vs. TBI).

At 3 days post-CCI, RT-qPCR revealed elevated pro-inflammatory (IL-1β, iNOS, IL-6, IFN-γ) and anti-inflammatory (IL-4, IL-10, IL-13, Arg-1) factors in TBI and DT+TBI groups vs. Sham (*P* < 0.05, *n* = 6). However, DT+TBI mice showed reduced pro-inflammatory expression (Figures 3A–D) and enhanced anti-inflammatory markers (Figures 3E–H) compared to TBI-alone mice (*P* < 0.05), indicating myeloid depletion skews the immune microenvironment toward an anti-inflammatory state during acute CCI.

### Myeloid depletion alters pro/anti-inflammatory polarization dynamics: FCM analysis

**Figure 4:**
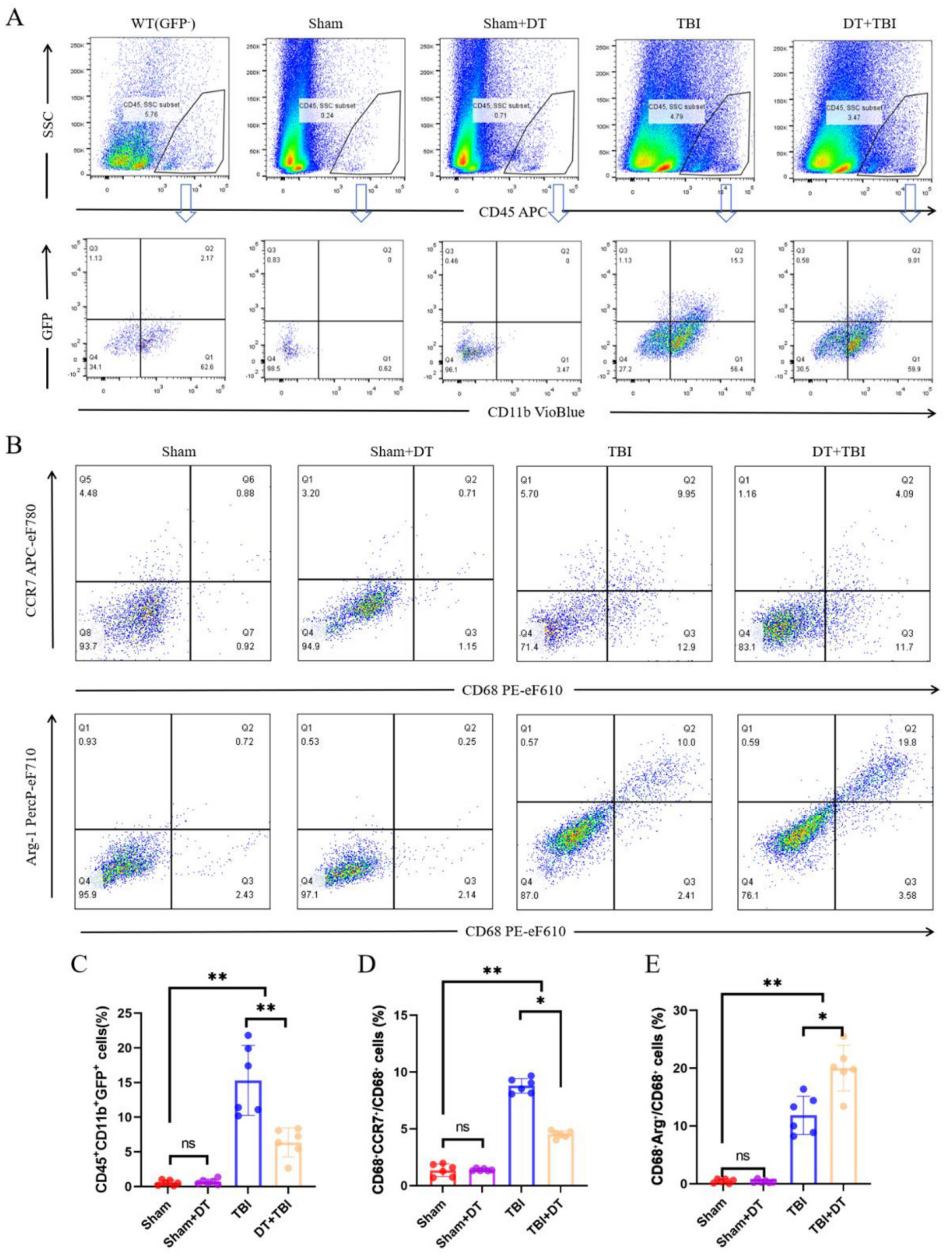
Myeloid depletion shifts pro/anti-inflammatory macrophage polarization dynamics post-CCI. **(A)**: Flow cytometry gating strategy: CD45^+^ cells were initially selected, followed by identification of CD11b^+^GFP^+^ myeloid cells. Wild-type (WT) mice served as negative controls. **(B)**: Representative flow cytometry pseudocolor plots of pro-inflammatory (CD68^+^CCR7^+^) and anti-inflammatory (CD68^+^Arg-1^+^) macrophage subsets. **(C)**: Quantification of CD45^+^CD11b^+^GFP^+^ cell proportions across groups (Sham, Sham+DT, TBI, DT+TBI). DT+TBI mice exhibited significant depletion versus TBI and Sham groups (*n* = 6; **P < 0.01). D-E: Proportions of pro-inflammatory **(D)** and anti-inflammatory **(E)** cells in total CD68^+^ polarized macrophages. DT + TBI mice showed reduced pro-inflammatory and increased anti-inflammatory polarization versus TBI mice (*n* = 6; *P < 0.05). Data are mean ± SEM.

The pro/anti-inflammatory polarization phenotypes of macrophages and microglia play an important role in controlling inflammatory responses following traumatic brain injury (Chen et al., 2018). To assess GFP^+^ cells and local pro-inflammatory and anti-inflammatory polarization seven days post-CCI, flow cytometry was performed using a combination of antibodies targeting CD45, CD11b, CD68, CCR7, Arg-1, and GFP. CD45^+^ cells were initially gated, followed by the identification of CD11b^+^GFP^+^ cells, with wild-type (WT) mice serving as negative controls. CD68^+^CCR7^+^ cells were defined as pro-inflammatory macrophages, and CD68^+^Arg-1^+^ cells were defined as anti-inflammatory macrophages. Flow cytometry analysis revealed no statistically significant difference in the proportion of CD45^+^CD11b^+^GFP^+^ cells between the Sham+DT and Sham groups. However, the fraction of CD45^+^CD11b^+^GFP^+^ cells was considerably lower in the TBI and DT+TBI groups compared to the Sham group, with an even greater reduction observed in the DT+TBI group compared to the TBI group (Figures 4A, C *n* = 6, *P* < 0.01). These results further confirmed that DT injection effectively reduced the number of myeloid cells, consistent with the findings from previous depletion experiments. Additionally, we quantified the overall proportions of pro-inflammatory and anti-inflammatory macrophages. The results showed no statistically significant differences in pro-inflammatory or anti-inflammatory proportions between the Sham+DT and Sham groups. However, both the TBI and DT+TBI groups had considerably higher numbers of pro-inflammatory and anti-inflammatory macrophages than the Sham group. Notably, the DT+TBI group revealed a large drop in CCR7-high (pro-inflammatory–associated) myeloid cells proportion and a considerable increase in Arg1-high (phagolysosomal/repair-associated) myeloid cells proportion when compared to the TBI group (Figures 4B, D–E, *P* < 0.05). These findings indicate that myeloid cell depletion inhibits pro-inflammatory polarization while promoting anti-inflammatory polarization during the early stage of TBI.

### Effect of myeloid depletion on microglia polarization phenotype of pro/anti-inflammatory: IHF detection

**Figure 5:**
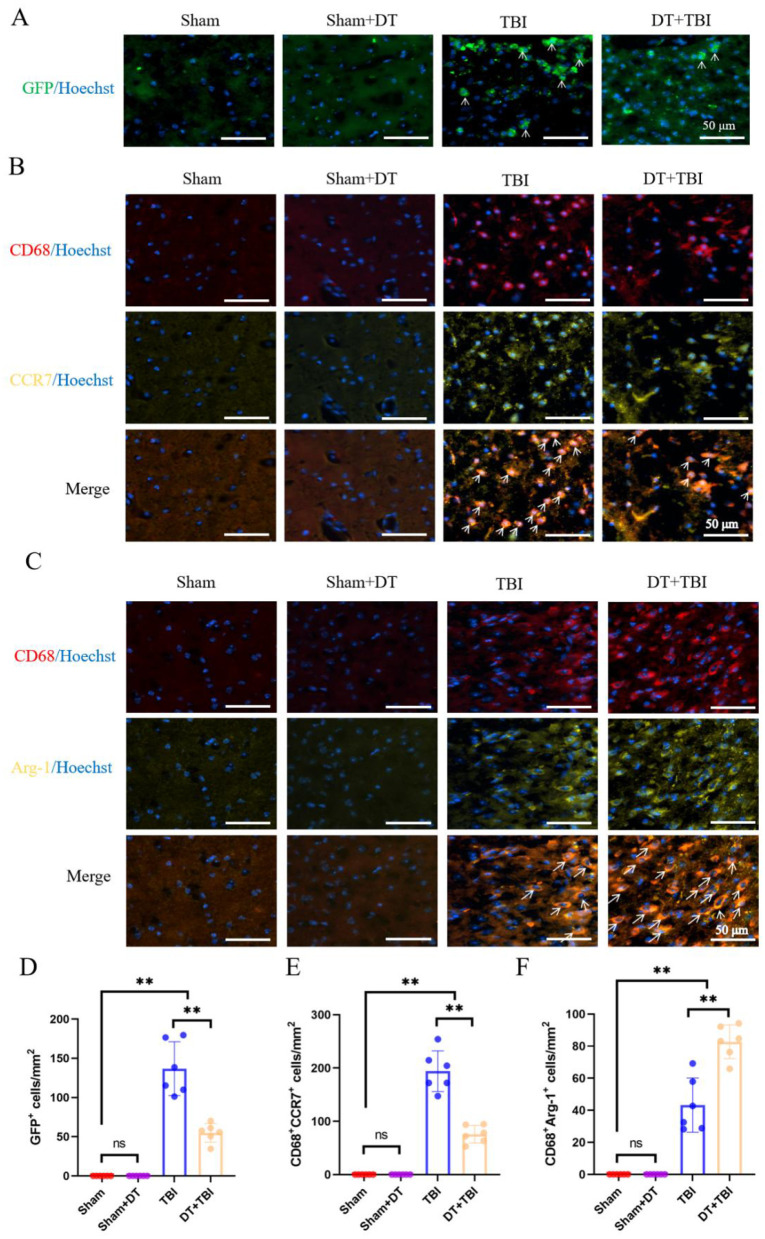
IHF validation of myeloid depletion effects on pro/anti-inflammatory polarization post-CCI. **(A)**: Representative IHF images of GFP^+^ myeloid cells (green) in brain sections. GFP^+^ cells were absent in Sham and Sham + DT groups but detected in TBI and DT+TBI groups, with significant reduction in DT+TBI versus TBI (*n* = 6; **P < 0.01). Nuclei were counterstained with Hoechst (blue). **(B,C)**: Representative multiplex IHF images of pro-inflammatory **(B)** and anti-inflammatory **(C)** macrophage markers in the peri-lesion area. To enhance specificity and clarity, the fluorophore for CCR7 and Arg-1 was changed from purple to yellow. Macrophages are identified by CD68 (red). Pro-inflammatory macrophages are defined as CD68^+^CCR7^+^ cells (orange, indicated by arrows). Anti-inflammatory macrophages are defined as CD68^+^Arg-1^+^ cells (orange, indicated by arrows). Note the minimal presence of positive cells in Sham and Sham + DT groups. **(D–F)**: Quantification of GFP^+^ cells **(D)**, pro-inflammatory **(E)**, and anti-inflammatory **(F)** macrophage proportions. DT + TBI mice exhibited reduced pro-inflammatory and increased anti-inflammatory polarization versus TBI (*n* = 6; **P < 0.01). Data are mean ± SEM; *P < 0.05, **P < 0.01 (DT + TBI vs. TBI).

To validate the impact of myeloid cell decrease on pro/anti-inflammatory polarization phenotypes seven days after TBI, IHF was used to confirm the flow cytometry data. CD68^+^CCR7^+^ cells were identified as pro-inflammatory macrophages, CD68^+^Arg-1^+^ cells as anti-inflammatory macrophages, and GFP^+^ cells as myeloid cells. Immunofluorescence results revealed that GFP^+^ cells were nearly absent in the Sham and Sham + DT groups, while GFP^+^ cells were detected in both the TBI and DT + TBI groups. Notably, the DT+TBI group exhibited a significant reduction in GFP^+^ cells compared to the TBI group (Figures 5A, D, *P* < 0.01), consistent with previous depletion experiments and flow cytometry findings. Although DT administration effectively depletes Lyz2^+^ myeloid cells, a small number of GFP^+^ cells may persist in the TBI + DT group due to incomplete depletion or repopulation from progenitor cells, as shown in the depletion kinetics (Figure 2). Additionally, some GFP^+^ cells may represent newly infiltrated monocytes that differentiated after the DT clearance period. In the same way, pro-inflammatory (Figure 5B) and anti-inflammatory (Figure 5C) cells were barely noticeable in the Sham + DT and Sham groups but were considerably more prevalent in the TBI and DT + TBI groups. The DT + TBI group showed a large drop in pro-inflammatory cells and a significant rise in anti-inflammatory cells when compared to the TBI group (Figure 5E, F, *P* < 0.01). These results suggest that myeloid cell depletion inhibits pro-inflammatory polarization, promotes anti-inflammatory polarization, and attenuates the inflammatory response during the early stage of CCI, thereby exerting a beneficial effect on recovery.

### Chronic behavioral deficits following myeloid depletion

Prior to injury, mice underwent pre-training in the water maze to exclude those with poor swimming ability or performance. Post-TBI, behavioral tests were administered in the following sequence: the open field test on day 21, the novel object recognition test on day 22, and the Morris water maze test from days 23 to 28.

**Figure 6:**
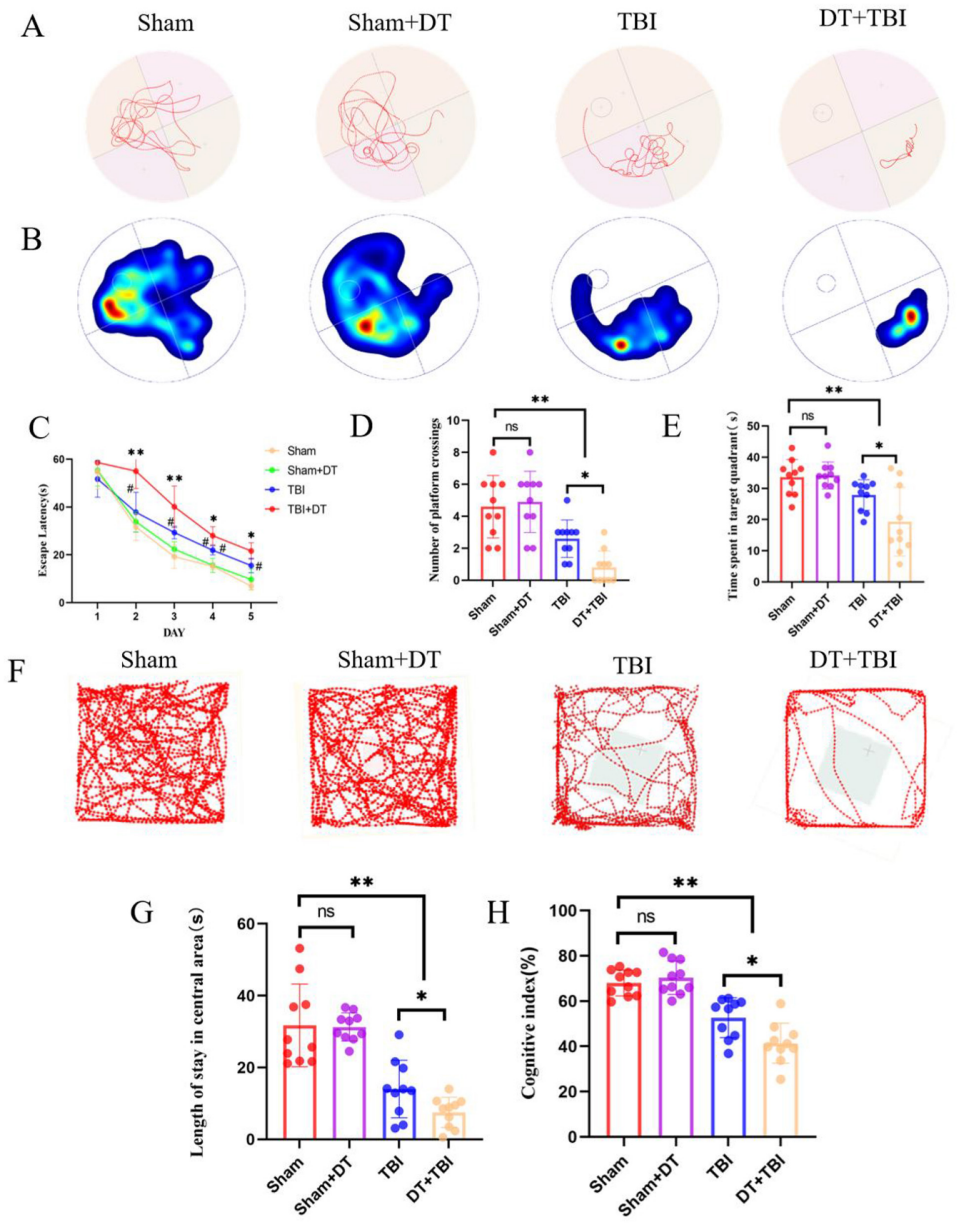
Myeloid depletion exacerbates chronic behavioral and cognitive deficits in CCI mice. **(A)**: Representative swimming trajectories in the Morris water maze during the probe trial (days 23–28 post-CCI). **(B)**: Heatmap depicting spatial preference for the target quadrant. **(C–E)**: Quantification of escape latency (*P < 0.05 compared with the TBI group, #*P* < 0.05 compared with the sham group) **(C)**, platform crossings **(D)**, and time spent in the target quadrant **(E)**. DT+TBI mice exhibited prolonged latency, fewer crossings, and reduced target quadrant time versus TBI and Sham groups (*n* = 6; *P < 0.05). **(F)**: Representative movement trajectories in the open field test (day 21 post-CCI). **(G)**: Time spent in the center zone during the open field test. DT + TBI mice showed reduced center exploration versus TBI and Sham groups (*P < 0.05). **(H)**: Statistical graph of the cognitive index. (*n* = 6, *P < 0.05, **P < 0.01).

The representative swimming trajectories and a heatmap of spatial preference are shown for each group (Figures 6A, B). In the Morris water maze test, the escape latency periods demonstrated a progressive decrease across training days in all groups, indicating gradual learning improvement. No statistically significant difference was observed between the Sham + DT and Sham groups. However, both the TBI and DT + TBI groups exhibited significantly longer escape latencies compared to the Sham group, with the DT + TBI group showing even longer latencies than the TBI group (Figure 6C, *P* < 0.05). Similarly, there was no significant difference in time spent in the target quadrant or at platform crossings between the Sham + DT and Sham groups. The TBI and DT + TBI groups had considerably fewer platform crossings and spent less time in the target quadrant than the Sham group, with the DT + TBI group doing worse than the TBI group (Figures 6D, E, *P* < 0.05). In the open field test, representative movement trajectories are shown in Figure 6F. There was no discernible difference between the Sham + DT and Sham groups in the open field test. However, both the TBI and DT + TBI groups spent significantly less time in the center of the open field compared to the Sham group, with the DT+TBI group showing even less time than the TBI group (Figure 6G, *P* < 0.05). The novel object recognition test, which evaluates cognitive memory in mice, was analyzed using the recognition index (RI), calculated as RI = (time spent exploring the novel object)/(time spent exploring the novel object + time spent exploring the familiar object) × 100% (Rashno et al., 2020). The results revealed no significant difference between the Sham + DT and Sham groups. In contrast to the Sham group, the TBI and DT + TBI groups displayed noticeably lower recognition indices; the DT + TBI group's index was even lower than the TBI group's (Figure 6H, *P* < 0.05). These findings suggest that myeloid cell depletion following CCI significantly impairs cognitive memory function in the chronic phase, highlighting the critical role of myeloid cells in maintaining cognitive memory during this period.

### Myeloid depletion exacerbates chronic histopathology

**Figure 7:**
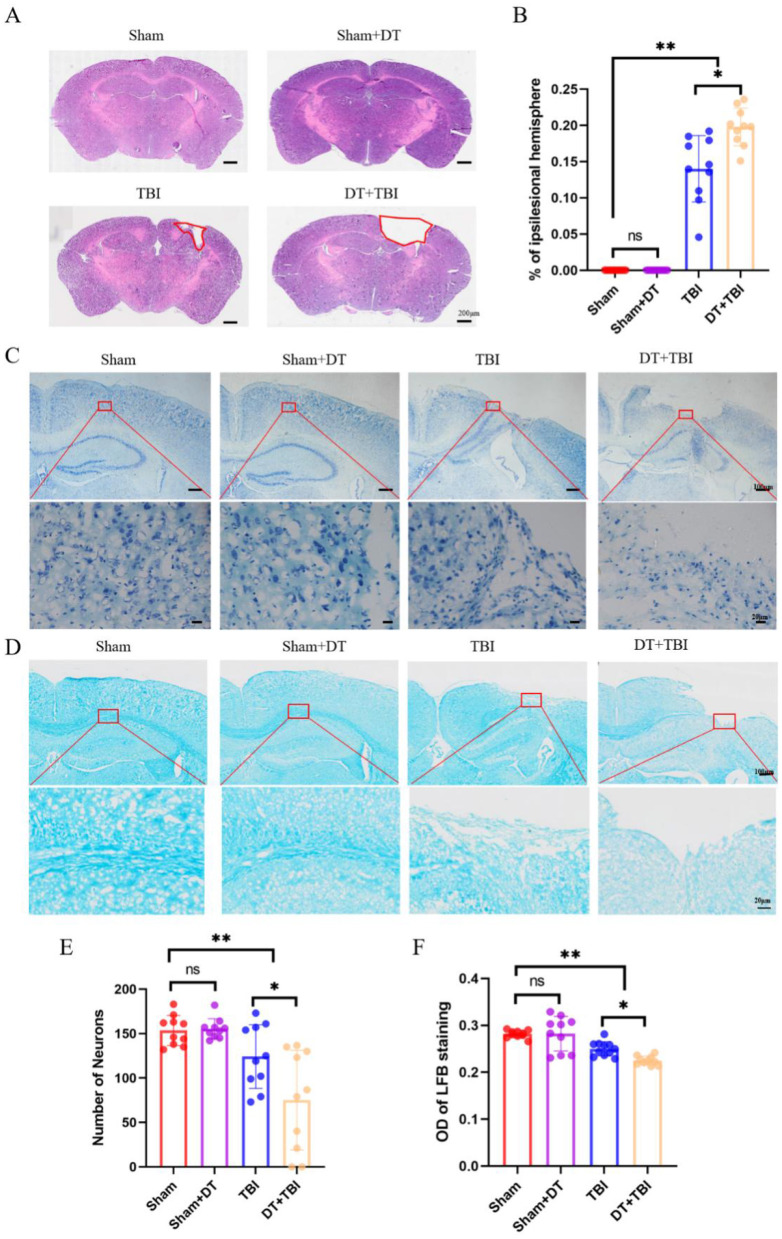
Myeloid depletion exacerbates chronic histopathological damage in CCI mice. **(A)**: Representative hematoxylin and eosin (HE)-stained brain sections showing tissue damage (dashed outlines) in the ipsilateral hemisphere 4 weeks post-CCI. **(B)**: Quantification of tissue loss. DT + TBI mice exhibited larger lesion volumes versus TBI mice (*n* = 6; *P < 0.05). **(C)**: Nissl-stained sections (cresyl violet) demonstrating neuronal survival in cortex. **(D)**: Luxol fast blue (LFB)-stained sections highlighting myelinated fibers (blue) in the hippocampal CA1 region. **(E)**: Quantification of surviving neurons in Nissl-stained sections. DT + TBI mice showed reduced neuronal counts versus TBI and Sham groups (*P < 0.05). **(F)**: Relative optical density (OD) of LFB staining, reflecting demyelination severity. DT + TBI mice displayed lower OD values versus TBI and Sham groups (*P < 0.05). Data are mean ± SEM.

To evaluate the impact of myeloid cell depletion on histopathological outcomes following CCI, brain tissue samples were collected 4 weeks post-injury and analyzed using hematoxylin and eosin (HE) staining. The area of tissue damage was quantified by averaging measurements from three consecutive sections at the same distance from the bregma. Tissue loss in the ipsilateral hemisphere was calculated as a percentage using the formula (contralateral hemisphere area—ipsilateral hemisphere area)/(contralateral hemisphere area)) 100% (Chen et al., 2023). The proportion of damaged region in the DT+TBI group was significantly higher than in the TBI group, according to statistical analysis (Figures 7A, B, *P* < 0.05), indicating that myeloid cell depletion exacerbates tissue damage in the chronic phase of CCI.

To investigate the impact of myeloid cell depletion on neuronal survival and demyelination in CCI mice, brain tissue sections were collected 4 weeks post-injury and subjected to Nissl staining (Figure 7C) and LFB staining (Figure 7D), respectively. Three consecutive sections at the same distance from the bregma were selected, and neurons in the same region were counted and averaged for statistical analysis. The findings showed that there was no discernible difference between the Sham+DT and Sham groups' numbers of surviving neurons. However, both the TBI and DT+TBI groups exhibited a significant reduction in surviving neurons compared to the Sham group, with the DT+TBI group showing even fewer neurons than the TBI group (Figure 7E, *P* < 0.05). These findings suggest that myeloid cell depletion reduces neuronal survival in the chronic phase of CCI.

To assess demyelination at the injury site 4 weeks post-CCI, three consecutive brain sections at the same distance from the bregma were analyzed. The CA1 region of the hippocampus was selected for relative OD value calculation using ImageJ software (Zhou et al., 2022), with measurements averaged across the three sections. There was no discernible difference between the Sham + DT and Sham groups, according to statistical analysis. But when compared to the Sham group, the relative OD values were considerably lower in the TBI and DT+TBI groups, with the DT+TBI group displaying even lower values than the TBI group (Figure 7F, *P* < 0.05). These results indicate significant demyelination in CCI mice, which was further exacerbated in the DT + TBI group. Collectively, these findings suggest that myeloid cell depletion significantly impairs histopathological recovery in the chronic phase of CCI.

## Discussion

Traumatic brain injury (TBI) represents a complex neuropathological condition in which the interplay between acute neuroinflammation and chronic repair mechanisms dictates long-term outcomes (Kalra et al., 2022). In this study, we demonstrate that targeted depletion of Lyz2^+^ myeloid cells during the acute phase of TBI exerts a dichotomous effect: it effectively suppresses pro-inflammatory responses and promotes anti-inflammatory polarization in the short term but paradoxically exacerbates chronic neurological deficits and histopathological damage. These findings contribute to a growing body of evidence challenging the traditional view of neuroinflammation as a uniformly detrimental process, instead highlighting its dual role in both injury propagation and recovery (Jassam et al., 2017). The transient suppression of Lyz2^+^ myeloid cells (primarily peripheral macrophages and microglia) aligns with studies emphasizing the importance of myeloid-derived signals in modulating the inflammatory cascade (Yamasaki et al., 2014). However, the long-term consequences of this intervention underscore the necessity of a balanced immune response, wherein acute inflammation serves as a scaffold for subsequent repair processes.

The acute anti-inflammatory effects observed in DT+TBI mice, characterized by reduced pro-inflammatory cytokines (IL-1β, iNOS, IL-6, IFN-γ) and enhanced anti-inflammatory mediators (IL-4, IL-10, IL-13, Arg-1), are consistent with prior work demonstrating that M1/M2 macrophage polarization exacerbates secondary injury through oxidative stress and blood-brain barrier disruption (Ruan et al., 2022). For example, IL-6 and IFN-γ have been implicated in amplifying neurotoxicity by activating glial cells and recruiting additional immune infiltrates (Jeannin et al., 2011). Conversely, the upregulation of M2-associated markers, such as Arg-1 and IL-10, aligns with their established roles in promoting tissue repair and resolving inflammation (Liu et al., 2020). These findings mirror observations in spinal cord injury models, where early M2 polarization enhances axonal sprouting and angiogenesis (Peng et al., 2021). However, the chronic deterioration in DT+TBI mice, manifested as impaired cognitive function, increased tissue loss, and reduced neuronal survival, suggests that the acute suppression of myeloid cells disrupts critical reparative pathways. This paradox echoes recent studies showing that prolonged M2 dominance can impair synaptic plasticity by suppressing inflammatory signals required for glial activation and debris clearance (Zheng et al., 2023; Kodali et al., 2021).

The dichotomy between acute benefits and chronic deficits may stem from the depletion of distinct myeloid subsets with temporally specialized functions. For instance, peripherally derived macrophages, which infiltrate the CNS within days post-TBI, are essential for phagocytosing cellular debris and secreting growth factors like TGF-β1 and VEGF, which facilitate glial scar formation and angiogenesis (Wang et al., 2013). Their transient elimination during the acute phase, as achieved in our model, likely delays the resolution of inflammation and compromises tissue remodeling. This hypothesis is supported by our histopathological findings, which revealed larger lesion volumes and exacerbated demyelination in DT+TBI mice. Consistent with parallel studies (Gitik et al., 2023; Wang et al., 2021), these findings indicate that inhibition of macrophage polarization and depletion of macrophage surface proteins impair both axonal regeneration and remyelination. Furthermore, the near-absence of GFP^+^ cells in the brain but their persistence in peripheral tissues (Figure 1C) suggests that infiltrating macrophages, rather than resident microglia, play a predominant role in chronic recovery, a notion corroborated by lineage-tracing studies demonstrating that peripheral macrophages contribute disproportionately to wound healing and synaptic remodeling (Henry et al., 2024; Saber et al., 2017).

The temporal specificity of myeloid cell functions revealed here has critical implications for therapeutic development. Clinical trials targeting broad-spectrum anti-inflammatory pathways, such as glucocorticoids or TNF-α inhibitors, have largely failed to improve long-term TBI outcomes, often due to unintended suppression of reparative immune activity (Zhou et al., 2021; Radpour et al., 2023). Our data suggest that phased immunomodulation, suppressing M1 polarization acutely while later promoting M2 transitions, may offer a more nuanced approach. For example, timed administration of IL-4 or IL-13 to enhance M2 polarization during the subacute phase could complement early anti-inflammatory strategies, as proposed in ischemic stroke models (Li et al., 2021; Wang et al., 2024). However, the translational relevance of our findings must be interpreted cautiously. The Lyz2-IRES-DTREGFP model, while enabling precise myeloid depletion, does not fully recapitulate human myeloid heterogeneity, particularly the functional divergence between monocyte subsets (CD14^+^ vs. CD16^+^) observed in clinical TBI (Chen et al., 2020).

The behavioral and cognitive deficits observed in DT+TBI mice further emphasize the importance of myeloid cells in maintaining neural circuit integrity. The Morris water maze and novel object recognition tests revealed that myeloid depletion not only prolonged escape latencies but also reduced recognition indices, indicative of impaired spatial and episodic memory. These findings align with studies linking hippocampal neurogenesis and synaptic plasticity to macrophage-derived BDNF and IGF-1 (Pan et al., 2019; Deyama et al., 2022). The exacerbated demyelination and neuronal loss in DT+TBI mice further suggest that myeloid cells support oligodendrocyte progenitor cell (OPC) differentiation and myelin repair, possibly via PDGF-AA or CNTF secretion (Gudi et al., 2014; de Almeida et al., 2020). Conversely, the absence of these signals in myeloid-depleted mice may create a hostile microenvironment for remyelination, as seen in our optical density analyses of the hippocampal CA1 region.

The limitations of this study merit careful consideration. First, the restriction of DT administration to a single time window (days 1–3 post-CCI) prevents assessment of how delayed or prolonged myeloid depletion influences functional recovery. Future studies should employ inducible depletion systems to resolve temporal contributions of myeloid cells. Second, a major constraint is the inability to distinguish between resident microglia, infiltrating monocyte-derived macrophages, and CNS-associated macrophages (CAMs), such as those in perivascular, meningeal, and choroid plexus regions, within the Lyz2^+^GFP^+^ population. Although CAMs are long-lived, Lyz2-expressing cells and may participate in TBI responses, the absence of specific markers (e.g., CD206, LYVE1, Mrc1) or anatomical context in our flow cytometry and immunohistochemical data precludes subset-specific mechanistic attribution (Jiang et al., 2025a). Furthermore, bulk RT-qPCR analysis of perilesional tissue, while informative of general inflammation, cannot assign cytokine production to specific cellular subsets due to mixed contributions from GFP^+^ myeloid cells, resident microglia, astrocytes, and neurons. Consequently, the cellular origin of observed cytokine shifts remains unresolved. Additionally, although our statistical models incorporated sex as a biological variable and detected no significant effects on primary outcomes, the study may be underpowered to identify subtler sex-specific modulations of myeloid responses. Finally, while this model underscores myeloid importance in chronic recovery, it does not account for potential compensatory mechanisms, such as astrocyte-driven inflammation or adaptive immune activation, which may influence outcomes.

To address these limitations, future work would benefit from: (1) conditional depletion systems to probe temporal roles; (2) advanced cellular discrimination via reporter mice (e.g., CX3CR1^Gfp^/CCR2^Rfp^) (Cherchi et al., 2022) FACS-sorting (Barthelaix et al., 2025), spatial transcriptomics (Jiang et al., 2025b), or single-cell RNA sequencing (Jordão et al., 2019); (3) marker-based or anatomical dissection of CAMs vs. microglia/macrophages; and (4) larger cohorts designed explicitly to evaluate sex differences. Such approaches will more precisely delineate subset-specific functions and systemic immune dynamics following TBI.

In conclusion, this study elucidates the dual roles of Lyz2^+^ myeloid cells in TBI, revealing that their acute depletion attenuates neuroinflammation but disrupts chronic repair processes essential for functional recovery. These findings challenge the simplistic view of inflammation as an adversary in TBI and advocate for therapeutic strategies that harmonize acute immunomodulation with the preservation of reparative immune functions. Future research should prioritize temporally and subset-specific interventions, leveraging advances in genetic targeting and multimodal immunophenotyping to refine TBI therapeutics. By bridging the gap between acute neuroprotection and chronic recovery, such approaches may finally unlock the translational potential of immunomodulation in TBI management.

## Data Availability

The original contributions presented in the study are included in the article/supplementary material, further inquiries can be directed to the corresponding author.
